# The relationship between lifestyle and serum neurofilament light protein in Huntington’s disease

**DOI:** 10.1002/brb3.1578

**Published:** 2020-03-17

**Authors:** Travis Cruickshank, Danielle Bartlett, Andrew Govus, Anthony Hannan, Wei‐Peng Teo, Sarah Mason, Johnny Lo, Mel Ziman

**Affiliations:** ^1^ School of Medical and Health Sciences Edith Cowan University Perth WA Australia; ^2^ Perron Institute for Neurological and Translational Science Perth WA Australia; ^3^ Department of Rehabilitation, Nutrition and Sport School of Allied Health La Trobe University Melbourne VIC Australia; ^4^ The Florey Institute of Neuroscience and Mental Health University of Melbourne Parkville VIC Australia; ^5^ Institute for Physical Activity and Nutrition (IPAN) Deakin University Geelong VIC Australia; ^6^ Physical Education and Sports Science (PESS) Academic Group National Institute of Education Nanyang Technological University Nanyang Singapore; ^7^ John Van Geest Centre for Brain Repair University of Cambridge Cambridge UK; ^8^ School of Science Edith Cowan University Perth WA Australia; ^9^ School of Pathology and Laboratory Medicine University Western Australia Perth WA Australia

**Keywords:** cardiorespiratory fitness, cognitive reserve, lifestyle, neurofilament light protein, social network

## Abstract

**Objectives:**

Serum neurofilament light protein (NfL) is a promising marker of disease onset and progression in Huntington's disease (HD). This study investigated associations between lifestyle factors and NfL levels in HD mutation carriers compared to healthy age‐ and sex‐matched controls.

**Materials and Methods:**

Participants included 29 HD mutation carriers and 15 healthy controls. Associations between serum NfL concentrations and lifestyle factors, including cardiorespiratory fitness, social network size and diversity, physical activity, cognitive reserve, smoking status, and alcohol consumption, were examined using a stepwise multivariable linear regression model.

**Results:**

Higher NfL levels were associated with lower cognitive reserve, social network size and diversity and cardiorespiratory fitness in HD mutation carriers. Group × lifestyle factor effects were observed between lower serum NfL levels and a greater social network diversity.

**Conclusion:**

These findings highlight a relationship between lifestyle factors and NfL levels in HD mutations carriers; however, longitudinal studies are required to confirm if these observed relationships persist over time.

## Abstract 2

Serum neurofilament light protein (NfL) is a promising marker of disease onset and progression in Huntington’s disease; however, it is not yet known how lifestyle factors may impact on NfL levels. Here, we report that lifestyle factors, particularly cognitive reserve, cardiorespiratory fitness and social network size and diversity, are significantly associated with NfL levels.

## 1 INTRODUCTION

Neurofilament light protein (NfL) is considered to be a biochemical marker of neuronal damage and shows promise as a measure of disease onset and progression in individuals with Huntington's disease (HD) (Byrne et al., 2017). NfL increases with advancing disease severity in individuals with HD (Byrne et al., 2017). Significant longitudinal associations have also been documented between increased NfL levels, striatal volume loss, and cognitive impairments in individuals HD (Johnson et al., 2018). These findings highlight the potential utility of NfL as a prognostic biomarker of disease onset and progression.

Lifestyle factors have been reported to influence the onset and rate of neurological and clinical deterioration in individuals with HD (Wexler et al., 2004). Significant associations have been reported between physical inactivity, smoking, higher caffeine and alcohol consumption and reduced dairy consumption and earlier onset of motor signs in HD patients (Marder, Gu, & Eberly, 2013; Schultz et al., 2017; Simonin et al., 2013; Trembath et al., 2010). Two independent longitudinal investigations have further shown that greater cognitive reserve, education, and body mass index are associated with a reduced rate of gray matter volume loss in striatal brain structures and slower deterioration of clinical decline, particularly function, motor, and cognitive abilities in individuals with premanifest HD (Bonner‐Jackson et al., 2013; Burg et al., 2017; Garcia‐Gorro et al., 2019; López‐Sendón et al., 2011; Martinez‐Horta et al., 2018). Together, these findings suggest that lifestyle factors influence the onset and progression of HD. The effects of lifestyle factors on NfL have not yet been investigated in individuals with HD, in relation to healthy controls. Considering that NfL is a potential biological readout of disease progression (Byrne et al., 2017), it is plausible that lifestyle factors influence levels of NfL and therefore warrant investigation, particularly as NfL has been considered as a biological measure for clinical trials.

This exploratory study examined associations between lifestyle factors and NfL levels in HD mutation carriers and healthy controls.

## 2 MATERIALS AND METHODS

### 2.1 Participants

Twenty‐nine HD mutation carriers and 15 healthy age‐ and gender‐matched controls were recruited for this study. Inclusion criteria for HD mutation carriers comprised disease‐specific measures, including a cytosine–adenine–guanine (CAG) repeat length ≥39, a diagnostic confidence score of ≤2, a total functional capacity (TFC) score of 13 (Reilmann, Leavitt, & Ross, 2014) on the Unified Huntington's Disease Rating Scale (UHDRS) and a scaled CAG‐age product (CAPs) score of ≥0.67 (CAPs = (CAGn‐33.66)/432.3326) (Zhang et al.&, 2011). Individuals were excluded if they had known musculoskeletal, metabolic, endocrine, immunological, cardiovascular, and sleep disorder comorbidities. Inclusion and exclusion criteria for healthy controls were as follows: no family history of HD, no known neurological, musculoskeletal, metabolic, endocrine, immunological, cardiovascular, or sleep conditions. Anxiety and depression symptomatology were assessed using the Hospital Anxiety and Depression Scale (HADS).

### 2.2 Study approval, registration and patient consent

This study was approved by North Metropolitan Area Mental Health Service, Edith Cowan University, Monash University and Deakin University Human Research Ethics Committees. Written informed consent was provided by all participants.

### 2.3 Serum NfL concentrations

Blood was collected via venepuncture into 8‐ml serum separation tubes (SSTs; Greiner Bio‐one). SSTs were centrifuged at 1,600 *g* for 10 min, divided into 500 μl aliquots and stored at −80°C. Serum samples were then shipped on dry ice to Quanterix, Massachusetts, USA, for NfL quantification (pg/ml) with the single‐molecule array (SIMOA) HD‐1 Accelerator program using the single‐plex NfL Advantage kit. Samples were analyzed in duplicate using the same batch of reagents. The intra‐assay coefficient of variation was 0.055.

### 2.4 Lifestyle factors

Lifestyle factors included measures of habitual physical activity, cognitive reserve, smoking status, and alcohol consumption, which are associated with measures of disease onset and progression (Bonner‐Jackson et al., 2013; Myers et al., 1991; Schultz et al., 2017; Trembath et al., 2010; Wexler et al., 2004). We also included measures of cardiorespiratory fitness and social network size and diversity, as these measures have been suggested to be environmental modifiers of healthy aging (Bickart, Wright, Dautoff, Dickerson, & Barrett, 2011; Kanai, Bahrami, Roylance, & Rees, 2012) and disease progression in Alzheimer's disease (Burns et al., 2008) and multiple sclerosis( Motl et al., 2015) patients.

Habitual physical activity including structured exercise, leisure, and housework activities over the last 12 months were retrospectively examined using the Minnesota Leisure Time Physical Activity Questionnaire (Pereira et al., 1997) and Compendia of Physical Activities database (Ainsworth et al., 2011, 1993). Cognitive reserve was calculated using values obtained from the National Adult Reading Test (Nelson & Willison, 1991), reported years of education, occupation status (O'Driscoll, Kalliath, Gudykunst & Kim, 2007), and the cognitive activity scale (Wilson et al., 1999, 2002). Values from these measures were converted to *z* scores and summated to generate a composite score for a cognitive reserve model, as mentioned in previous studies (Bonner‐Jackson et al., 2013; Tucker & Stern, 2011). Body mass index was calculated using body mass and height measurements collected using dual‐energy x‐ray absorptiometry and a stadiometer (Thompson et al., 2013). Smoking status (packets per week) and alcohol consumption (standard drinks consumed per week) were assessed via a custom questionnaire (Appendix S1). Cardiorespiratory fitness (heart rate and maximal oxygen consumption) was measured using a graded exercise test performed on a bicycle ergometer. Social network size and diversity was evaluated using components of the Social Network Index (SNI) (Cohen, Doyle, Skoner, Rabin, & Gwaltney, 1997). Social network diversity reflects the number of social roles that an individual has regular contact with (at least once every 2 weeks; spouse, parent, child, close friend, neighbor, employee, student, etc.). Social network size reflects the total number of people with whom the individual has regular contact with (at least once every 2 weeks). The online social network of individuals was also assessed via the quantification of Facebook friends (Dunbar, 2016; Kanai et al., 2012) (for more information see Appendix S1).

### 2.5 Statistical analyses

Descriptive statistics were presented as mean and standard deviation for normally distributed data, while median and interquartile range were presented for non‐normal data. Normality assumptions were tested using a Shapiro–Wilk test. Independent *t* tests were used to compare continuous variables between HD mutation carriers and healthy control groups when data were normally distributed. Mann–Whitney *U* tests were applied when data were not normally distributed. Chi‐square tests were used to analyze categorical variables. Linear regression analysis was performed to test the associations between NfL concentrations (pg/ml) and each of the lifestyle factors within each group. Factors that were initially significant were then entered into a general linear model to formally compare the lifestyle factors between the HD mutation carriers and healthy controls by examining the group × lifestyle interaction. Age and sex were adjusted for in all regression models. Log‐transformation was applied to NfL concentrations, and log, square root, and fourth root transformations were applied as appropriate to lifestyle factors that departed from normality. As this was an exploratory study, no correction was applied to adjust for multiple comparisons. Two‐tailed statistical significance was set at *p* ≤ .05. Statistical analyses were performed using IBM SPPS Statistics Version 23.

## 3 RESULTS

### 3.1 Participant demographics

There were no differences in age and sex between HD mutation carriers and healthy control groups (Table 1 and Table S1). Neither group had clinically relevant anxiety or depression symptomatology as identified by the HADS.

**Table 1:** Demographic and lifestyle factor data for HD mutation carriers and healthy controls

Outcomes	HD mutation carriers (*n* = 29)	Healthy controls (*n* = 15)	*p*‐Value
Demographics			
Age^a^	44.6 ± 11.8	44.5 ± 10.1	.981
Female^b^	19 (65.0%)	10 (67.0%)	.939
CAGn	43.0 ± 3.0	N/A	N/A
CAPs	0.9 ± 0.2	N/A	N/A
UHDRS‐TMS	4.7 ± 7.6	N/A	N/A
Total functional capacity	13.0 ± 0.0	N/A	N/A
Diagnostic confidence level	0.4 ± 0.7	N/A	N/A
NART^c^	119.5 ± 4.6	121.4 ± 2.6	.008*
Body mass index^a^	25.9 ± 3.1	26.8 ± 2.3	.363
HADS‐Total^a^	7.8 ± 5.3	7.4 ± 4.7	.803
HADS‐Anxiety^a^	5.3 ± 3.6	5.9 ± 3.3	.646
HADS‐Depression^c^	2.0 ± 3.0	1.5 ± 3.0	.256
Biological outcome			
NfL (pg/ml)^c^	12.9 ± 15.1	9.6 ± 12.1	.245
Lifestyle factors			
VO_2_ max (ml kg^−1^ min^−1^)^c^	28.2 ± 7.1	33.0 ± 8.6	.025*
Minnesota Leisure Time Physical Activity Questionnaire			
Exercise (MET Min)^c^	10,929.0 ± 32,124.4	22,542.5 ± 106,542.0	.376
Recreation (MET Min)^c^	53,392.6 ± 128,084.7	88,541.4 ± 137,209.9	.990
Housework (MET Min)^c^	8,232.8 ± 36,141.9	13,437.1 ± 24,029.0	.970
Total activity (MET Min)^c^	137,834.4 ± 221,827.1	171,583.6 ± 239,230.6	.701
Cognitive reserve			
Education (Number of years; *n* = 12)^a^	16.6 ± 5.8	15.3 ± 2.5	.217
Occupational complexity^c^	5.0 ± 5.0	8.0 ± 1.0	.020*
Cognitive Leisure Scale^a^	30.6 ± 5.1	29.0 ± 4.2	.307
Cognitive reserve a	−0.1 ± 0.58	1.8 ± 0.6	.334
Social network			
Number of high contact roles^a^	5.8 ± 1.6	8.7 ± 1.9	<.001*
Number of social contacts^c^	17.5 ± 13.0	26.0 ± 19.0	.007*
Embedded social networks^c^	2.0 ± 2.0	2.0 ± 1.0	.032*
Facebook friends^c^	41.0 ± 136.5	120.0 ± 356.0	.280
Smoking status^b^	7 (24.1%)	0 (0%)	.041*
Alcohol consumption^c^	1.0 ± 6.0	2.0 ± 4.0	1.000

Abbreviations: CAGn, number of cytosine–adenine–guanine repeats; CAPs, scaled CAG‐age product score (CAPs, (CAGn‐33.66)/432.3326); HADS, Hospital Anxiety and Depression Scale; HD, Huntington's disease; MET, metabolic equivalent; NART, National Adult Reading Test; NfL, neurofilament light protein; UHDRS‐TMS, Unified Huntington's Disease Rating Scale–Total Motor Score; VO_2_ max, maximal oxygen consumption.

a Data were normally distributed; mean ± *SD* are presented; independent *t* test was applied;

b data are categorical; *n* (%) are presented; chi‐square test was applied;

c data were non‐normally distributed; median ± IQR are presented; Mann–Whitney *U* test was applied.

* Indicates *P* < .05.

### 3.2 Serum Nfl concentrations

There were no differences in the concentrations of NfL between HD mutation carriers and healthy control groups (Table 1).

### 3.3 Lifestyle outcomes

A number of differences were observed for lifestyle factors between groups (Table 1). A greater number of smoking pack years was seen in HD mutation carriers than healthy controls. Compared with healthy controls, HD mutation carriers had lower occupational diversity and cardiorespiratory fitness. HD mutation carriers also had fewer high contact roles, social contacts, and embedded social networks. There were no other differences for lifestyle factors between groups.

### 3.4 Associations between NfL levels and clinical and lifestyle factors in HD mutation carriers

The relationships between serum NfL concentration and diagnostic confidence score and CAP score were assessed. While no significant association was observed between diagnostic confidence score and NfL levels (*r*_s_ = .173, *p* = .370), significant positive associations were observed between CAP score and NfL levels (*r*_s_ = .609, *p* < .001), suggesting that individuals closer to diagnosis had a greater serum NfL concentration. Higher serum NfL was associated with lower cognitive reserve and lower number of high contact roles in HD mutation carriers (Table 2).

**Table 2:** Associations between log‐transformed NfL levels and lifestyle factors in HD mutation carriers and in healthy controls. Models are adjusted for gender and age

Predictor	Estimate (95% CI)	*p*‐Value	Model *R* ^2^
HD mutation carriers			
High contact roles	−0.18 (−0.3, −0.06)	.007**	.471
Cognitive reserve	−0.03 (−0.05, −0.00)	.024*	.395
Sports leisure (×10^5^)^#^	0.06 (−0.00, 0.120)	.057	.358
Exercise^a^	−0.03 (−0.06, 0.01)	.152	.316
Number of social contacts^a^	−0.41 (−0.97, 0.14)	.161	.325
VO_2_ max (ml kg^−1^ min^−1^)^a^ (×10)^#^	−0.21 (−0.54, 0.12)	.226	.299
Housework (×10^5^)	−0.17 (−0.58, 0.23)	.410	.276
Alcohol (×10)	0.11 (−0.17, 0.40)	.445	.461
Embedded social network^b^	−0.13 (−0.47, 0.21)	.465	.278
Number of facebook friends^a^	−0.07 (−0.35, 0.20)	.610	.286
Smoking status	0.08 (−0.41, 0.58)	.741	.259
Total physical activity^a^	0.00 (−0.03, 0.04)	.933	.256
Healthy controls			
VO_2_ max (ml kg^−1^ min^−1^)^a^ (×10)^#^	−0.46 (−0.84, −0.09)	.036*	.583
Number of facebook friends^a^	−0.18 (−0.45, 0.08)	.219	.659
Total physical activity^a^	−0.02 (−0.06, 0.02)	.280	.491
High contact roles	0.07 (−0.07, 0.20)	.360	.474
Cognitive reserve	0.01 (−0.01, 0.03)	.364	.474
Embedded social network^b^	0.34 (−0.54, 1.22)	.462	.459
Sports leisure (×10^5^)^#^	−0.12 (−0.48, 0.24)	.525	.452
Exercise^a^	−0.01 (−0.05, 0.03)	.584	.447
Alcohol (×10)^#^	−0.04 (−0.41, 0.33)	.830	.433
Number of social contacts^a^	0.13 (−1.21, 1.47)	.851	.433
Housework (×10^5^)^#^	0.03 (−0.32, 0.39)	.856	.433

Abbreviations: CI, confidence interval; HD, Huntington's disease; VO_2_ max, maximal oxygen consumption.

a Fourth root transformation was applied.

b Square root transformation was applied;

^#^ Exp (estimate) provides the change in the NFL for every unit (or 10^x^ for certain predictors) increase in the predictor.

* Significant at 5% level of significance;

** Significant at 1% level of significance.

### 3.5 Associations between NfL and lifestyle factors in healthy controls

Higher cardiorespiratory fitness was associated with lower NfL levels in healthy controls (Table 2 and Table S2). No other relevant associations were observed.

### 3.6 Differences in the associations between NfL and lifestyle factors in HD mutation carriers relative to healthy controls

Group × lifestyle factor effects were examined for number of high contact roles, cognitive reserve and cardiorespiratory fitness between healthy controls and HD mutation carriers via GLM. The models confirmed that differences in the number of high contact social roles (*p* = .039) and cardiorespiratory fitness (*p* = .029) in healthy controls compared to HD mutation carriers have a significant association with NfL levels. However, while a trend toward a difference in cognitive reserve between healthy controls and HD mutation carriers was observed, this was not significant (*p* = .055).

## 4 DISCUSSION

Neurofilament light protein represents a promising prognostic marker of disease onset and progression in HD mutation carriers (Byrne et al., 2017). Despite knowledge that lifestyle influences disease onset and progression (Bonner‐Jackson et al., 2013; Trembath et al., 2010; Wexler et al., 2004), no studies have investigated potential associations between known lifestyle factors and NfL levels in HD. We investigated associations between a myriad of lifestyle factors and NfL levels in HD mutation carriers and compared these associations to age‐ and sex‐matched heathy controls. We found significant associations between measures of cognitive reserve, social network size and diversity and NfL levels in HD mutation carriers and cardiorespiratory fitness and NfL levels in healthy controls. Tests of the group × lifestyle modifier effects confirmed that differences in social network size and diversity and cardiorespiratory fitness between HD mutation carriers and healthy controls were significantly associated with NfL levels.

Greater cognitive reserve and social network size and diversity were found to be associated with lower NfL levels in HD mutation carriers, with the strongest associations appearing between measures of social network size and diversity and NfL levels and differentiated between HD mutation carriers and healthy controls. Cardiorespiratory fitness was inversely related to NfL in controls, but such association was not observed in HD mutation carriers. To our knowledge, this is the first study reporting significant associations between lifestyle factors and NfL levels in HD and healthy individuals. The nature and mechanistic underpinnings of these associations are not known. It is possible that NfL levels could be indirectly influenced by the modulatory role of lifestyle factors on brain structures, which has been demonstrated in mouse models of HD and cross‐sectional investigations in patients, as well as other neurodegenerative disorders (Bonner‐Jackson et al., 2013; Dellen, Blakemore, Deacon, York, & Hannan, 2000; Trembath et al., 2010). It is also plausible that lifestyle factors have a direct influence on NfL levels; however, focused mechanistic studies are needed to confirm this supposition. Regardless of the nature of the associations, direct or indirect, additional longitudinal studies are needed to confirm whether the observed associations persist over time.

This study has several limitations which need to be considered when interpreting our findings. First, this was a cross‐sectional study of associations between serum NfL levels and lifestyle factors and therefore causation cannot be established. Second, this study included a small sample of HD mutation carriers and healthy controls. It is also possible that group differences in associations between lifestyle factors and NfL levels arose due to uneven participant numbers across the groups. It is important to note that participants included in the manuscript were close to clinical onset. It is therefore possible that the noted associations between lifestyle factors and serum NfL are reflective of disease stage as opposed to the protective effects of lifestyle factors on serum NfL. This study also did not include individuals with moderate or advanced HD, so it is not yet known whether the observed associations are representative of all individuals with HD. Future studies are needed to confirm whether the observed associations between lifestyle factors and serum NfL are indeed present or indicative of disease progression.

Despite these notable limitations, this study provides important, preliminary data to suggest a relationship between cognitive reserve and social network size and diversity and NfL levels in HD mutation carriers. This study also suggests a differential association between lifestyle factors and NfL levels in HD mutation carriers compared to healthy controls. These findings have important implications regarding the measurment of NfL in clinical studies, however further longitudinal investigations in larger cohorts of individuals with HD are warranted.

## CONFLICT OF INTEREST

All authors declare no conflicts of interest.

## AUTHOR CONTRIBUTIONS

T.C, D.B, and M.Z conceptualized and conducted the study. A.G and W‐P.T assisted with the collection and interpretation of lifestyle data. A.H and S.M contributed to the design, interpretation of study findings, and editing of the study manuscript. J.L entered and analyzed study data and assisted in the interpretation and writing of the study manuscript. T.C, D.B, and M.Z drafted and edited the study manuscript.

## Data Availability

The data that support the findings of this study are available on request from the corresponding author. The data are not publicly available due to privacy or ethical restrictions.
