# Supplementary material for: The relationship between lifestyle and serum neurofilament light protein in Huntington’s disease
Source: Brain Behav. 2020 Mar 17;10(5):e01578. doi: 10.1002/brb3.1578 (PMC7218250; doi:10.1002/brb3.1578)
Supplement: Supplementary file 1 [file BRB3-10-e01578-s001.docx]

**SUPPLEMENTARY DIGITAL CONTENT**

**MATERIALS AND METHODS**

**Environmental Factors**

*Smoking, Alcohol Consumption and Medication Use*

Information on smoking status, alcohol consumption and medication use was collected using a questionnaire (appended at the end of this document).

*Cardiorespiratory Fitness (VO_2_max)*

A graded exercise test was used to measure cardiorespiratory fitness. The test was performed on an electronically braked stationary bicycle ergometer (Velotron Dynafit Pro cycle ergometer, RacerMate Inc, WA, USA). Expired gas was collected continuously during each test using an automated breath-by-breath gas analysis system (Parvo TrueOne 2400, Parvo Medics, USA). Capillary blood was also sampled from the index finger of participants immediately before and one minute following testing and analysed for changes in blood lactate concentration using a handheld, automated blood lactate analyser (Lactate Pro 2, Arkray Inc., Kyoto, Japan). Before each test, participants pedalled for ten minutes at 80 W (males) or 60 W (females) at a cadence of 60 revolutions per minute (revs.min^-1^), followed by five minutes of seated rest. After one minute of unloaded cycling, the graded exercise test commenced at 80 W (males) and 60 W (females) and the workload was increased by 5 W each 15 seconds (i.e. 20 W each minute) until participants reached volitional exhaustion or could not maintain a pedal cadence greater than 40 revs.min^-1^. Strong verbal encouragement was provided to all participants throughout the test until the point of volitional exhaustion. Participants’ heart rate (HR; RS400, Polar Electro, Kempele, Finland) and rating of perceived exertion (RPE; using a 6-20 scale)^1^ were collected at each minute during the test. Peak oxygen uptake (VO_2_ peak) was calculated as the highest 15 second average oxygen value (VO_2_) observed during the last successfully completed stage.

*Physical Activity*

The Minnesota Leisure Time Physical Activity questionnaire requires participants to retrospectively report on time spent (monthly frequency and time spent [hr/min]) performing structured exercise (i.e. weight training), sports (i.e. tennis) and leisure (i.e. water skiing) and household activities (i.e. mowing the lawn) in the past year. Using data collected from this questionnaire, monthly and yearly standardised metabolic equivalent (MET) values were calculated for all reported activities^2,3^. MET values are reported in minutes for each activity, composites of related activities (exercise, sports and leisure and household activities) and yearly activities (sum of all reported activities).

*Cognitive Reserve*

Cognitive reserve was calculated as a composite of scores from assessments estimating premorbid intellectual level, education level, occupation status and cognitive leisure.

The National Adult Reading Test was used to assess the intellectual level of participants, with larger values indicating a higher intellectual level^4^. The education level of participants was also assessed by calculating total years of education.

Occupation status was determined using the Australian Standard Classification of Occupations (ASCO)^5^. Occupational status was rated on a scale of 1-9 (1 = Managers and Administrators, 2 = Professionals, 3 = Associated Professionals, 4 = Tradespersons and Related Workers, 5 = Advanced Clerical and Service Workers, 6 = Intermediate Clerical, Sales and Service Workers, 7 = Intermediate Production and Transport Workers, 8= Elementary Clerical, Sales and Service Workers, 9 = Labourers and Related Workers). Participants who reported that they were not in the workforce were given a score of 0. Occupational status scores were then reverse coded for inclusion as a composite score within the cognitive reserve model, with a greater score equating to a larger occupational complexity^6^.

Cognitive leisure was assessed in participants using a modified version of the cognitive activity scale proposed by Wilson et al^7,8^. This scale required participants to report the frequency (5 = every day or about every day, 4 = several times a week, 3 = several times a month, 2 = several times a year, 1 = once a year or less) in which participants perform the following cognitive leisure activities; viewing television, listening to the radio, reading magazines and newspapers, playing videogames, reading books, playing structured games, going to museums/plays/concerts, producing art, playing a musical instrument, producing non-artistic writing and attending lectures/talks/seminars. This scale has been shown to be useful for measuring retrospective cognitive leisure activities in patients with Alzheimer’s disease and multiple sclerosis^9^.

**RESULTS**

**Supplementary Table S1.** Demographic and clinical characteristics of individuals with premanifest and early manifest HD and healthy controls.

|  | **Premanifest (n=20)** | **Early Manifest (n=9)** | **Healthy Controls (n=15)** |
| --- | --- | --- | --- |
| Age^1^ | 44.3 ± 11.5 | 45.1 ± 13.0 | 44.5 ± 10.1 |
| Female^2^ | 12 (60.0%) | 7 (77.8%) | 10 (67.0%) |
| CAGn | 42.7 ± 2.6 | 43.7 ± 3.7 | - |
| CAPs | 0.8 ± 0.2 | 0.96 ± 0.22 | - |
| UHDRS-TMS | 0.4 ± 0.7 | 14.3 ± 7.1 | - |
| Total Functional Capacity | 13.0 ± 0.0 | 13.0 ± 0.0 | - |
| Diagnostic Confidence Level | 0.0 ± 0.0 | 1.3 ± 0.7 | - |

**Supplementary Table S2.** Associations between log-transformed NfL levels and lifestyle factors in individuals with premanifest and early manifest HD and healthy controls.

| **Predictor** | **Premanifest (n=20)** | | | **Early Manifest (n=9)** | | | **Healthy Controls (n=15)** | | |
| --- | --- | --- | --- | --- | --- | --- | --- | --- | --- |
|  | **Estimate^#^ (95% CI)** | ***p*-value** | **Model R^2^** | **Estimate^#^ (95% CI)** | ***p*-value** | **Model R^2^** | **Estimate^#^ (95% CI)** | ***p*-value** | **Model R^2^** |
| High Contact Roles | -0.29 (-0.48, -0.10) | 0.011* | 0.533 | -0.14 (-0.39, 0.12) | 0.349 | 0.222 | 0.07 (-0.07, 0.20) | 0.36 | 0.474 |
| Sports Leisure (×10^5^) | 0.06 (-0.00, 0.13) | 0.083 | 0.353 | -0.24 (-0.83, 0.35) | 0.461 | 0.163 | -0.12 (-0.48, 0.24) | 0.525 | 0.452 |
| Cognitive Reserve | -0.03 (-0.06, 0.00) | 0.100 | 0.341 | -0.05 (-0.10, 0.01) | 0.143 | 0.411 | 0.01 (-0.01, 0.03) | 0.364 | 0.474 |
| VO_2_ max (mL.kg^-1^.min^-1^)^2^ (×10) | -0.41 (-0.92, 0.10) | 0.139 | 0.318 | -0.09 (-0.61, 0.43) | 0.751 | 0.077 | -0.46 (-0.84, -0.09) | 0.036* | 0.583 |
| Alcohol (×10) | 0.21 (-0.09, 0.52) | 0.198 | 0.540 | -0.73 (-2.97, 1.51) | 0.552 | 0.127 | -0.04 (-0.41, 0.33) | 0.830 | 0.433 |
| Number of Social Contacts^2^ | -0.75 (-1.92, 0.42) | 0.234 | 0.306 | -0.29 (-1.08, 0.5) | 0.501 | 0.146 | 0.13 (-1.21, 1.47) | 0.851 | 0.433 |
| Exercise^2^ | -0.03 (-0.08, 0.02) | 0.236 | 0.283 | -0.04 (-0.10, 0.01) | 0.198 | 0.345 | -0.01 (-0.05, 0.03) | 0.584 | 0.447 |
| Body Mass Index | -0.04 (-0.13, 0.05) | 0.412 | 0.248 | -0.03 (-0.16, 0.10) | 0.669 | 0.094 | -0.02 (-0.40, 0.35) | 0.101 | 0.552 |
| Embedded Social Network^1^ | -0.29 (-1.08, 0.50) | 0.480 | 0.253 | -0.27 (-0.83, 0.29) | 0.386 | 0.201 | 0.34 (-0.54, 1.22) | 0.462 | 0.459 |
| Housework (×10^5^) | -0.17 (-0.65, 0.32) | 0.515 | 0.236 | 0.2 (-1.90, 2.29) | 0.860 | 0.063 | 0.03 (-0.32, 0.39) | 0.856 | 0.433 |
| Total Physical Activity^2^ | 0.01 (-0.04, 0.05) | 0.777 | 0.219 | -0.03 (-0.10, 0.04) | 0.447 | 0.169 | -0.02 (-0.06, 0.02) | 0.280 | 0.491 |
| Smoking Status | 0.09 (-0.59, 0.77) | 0.801 | 0.218 | 0.02 (-1.03, 1.06) | 0.976 | 0.056 | NA | NA | NA |
| Number of Facebook Friends ^2^ | 0.04 (-0.40, 0.47) | 0.878 | 0.220 | -0.63 (-0.73, -0.53) | 0.001** | 0.981 | -0.18 (-0.45, 0.08) | 0.219 | 0.659 |

* Significant at 5% level of significance; ** Significant at 1% level of significance; ^1^ Square root transformation was applied; ^2^ Fourth root transformation

was applied, CI = confidence interval, **^#^** Exp (estimate) provides the change in the NFL for every unit (or 10^x^ for certain predictors) increase in the predictor.

**REFERENCES**

1. Borg GA. Psychophysical bases of perceived exertion. *Med Sci Sports Exerc.* 1982;14(5):377-381.

2. Ainsworth BE, Haskell WL, Leon AS, et al. Compendium of Physical Activities. *Med Sci Sports Exerc.* 1993;25(1):71-80.

3. Ainsworth BE, Haskell WL, Whitt MC, et al. Compendium of Physical Activities: An update of activity codes and MET intensities. *Med Sci Sports Exerc.* 2000;32(9; SUPP/1):S498-S504.

4. Nelson HE, Willison J. *National Adult Reading Test (NART).* Nfer-Nelson Windsor; 1991.

5. O'Driscoll PT, Kalliath T, Gudykunst W, Kim Y. Australian Standard Classification of Occupations. *Psychology.*88:635-646.

6. Bonner-Jackson A, Long JD, Westervelt H, Tremont G, Aylward EH, Paulsen JS. Cognitive reserve and brain reserve in prodromal Huntington's disease. *Journal of the International Neuropsychological Society : JINS.* 2013;19(7):739-750.

7. Wilson RS, Bennett DA, Beckett LA, et al. Cognitive activity in older persons from a geographically defined population. *The Journals of Gerontology Series B: Psychological Sciences and Social Sciences.* 1999;54(3):P155-P160.

8. Wilson RS, De Leon CFM, Barnes LL, et al. Participation in cognitively stimulating activities and risk of incident Alzheimer disease. *JAMA.* 2002;287(6):742-748.

9. Sumowski JF, Wylie GR, Gonnella A, Chiaravalloti N, Deluca J. Premorbid cognitive leisure independently contributes to cognitive reserve in multiple sclerosis. *Neurology.* 2010;75(16):1428-1431.

**Social and Medication History**

The following questions are about your medication and smoking status and alcohol consumption at the time of your testing for the HEROs 2.0 study. Please complete this form and return it via email to [d.bartlett@ecu.edu.au](mailto:d.bartlett@ecu.edu.au) at your earliest convenience.

1. Do you smoke? Yes/No (if yes, please proceed to question 2. If no, please skip to question 4)
2. For how many years have you smoked? _________________
3. How many packs do you smoke per day? ________________
4. How many standard alcoholic beverages would you consume on an average week? _______
5. Are you on any medications? If so, please provide as much detail as possible in the table below:

| **Medication Name** | **Dose** | **Frequency** | **Start Date** (mm/dd/yyyy) | **End Date** (mm/dd/yyyy) | **Ongoing?** |
| --- | --- | --- | --- | --- | --- |
|  |  |  |  |  | Yes   No |
|  |  |  |  |  | Yes   No |
|  |  |  |  |  | Yes   No |
|  |  |  |  |  | Yes   No |
|  |  |  |  |  | Yes   No |
|  |  |  |  |  | Yes   No |

Thank you for taking the time to complete this form. Your participation in this study is very much appreciated.
